# A dataset of scientific dates from archaeological sites in eastern Africa spanning 5000 BCE to 1800 CE

**DOI:** 10.1038/s41597-025-05138-x

**Published:** 2025-05-16

**Authors:** Victor Iminjili, Alison Crowther, Michael T. Fisher, Andrea Kay, Patrick Roberts, Steve Goldstein, Nicole Boivin, Ricardo Fernandes

**Affiliations:** 1 Department of Archaeology, Max Planck Institute of Geoanthropology, Kahlaische Strasse 10, 07745 Jena, Germany; 2 Institute for Prehistory and Early History, University of Cologne, Weyertal 125, 50931 Cologne, Germany; 3 School of Social Science, The University of Queensland, St Lucia, QLD, 4072 Brisbane, Australia; 4 Department of Anthropology, University of Pittsburgh, 3302 WWPH, Pittsburgh, PA 15260 USA; 5 Griffith Sciences, Griffith University, Brisbane, Australia; 6 Department of Bioarchaeology, Faculty of Archaeology, University of Warsaw, Warszawa, 00-927 Poland; 7 Arne Faculty of Arts, Masaryk University, Nováka 1, 602 00, Brno-střed, Czech Republic; 8 Climate Change and History Research Initiative, Princeton University, Princeton, NJ 08542 USA

**Keywords:** Palaeoecology, Environmental sciences

## Abstract

Large collections of archaeological spatiotemporal data can reveal past cultural and demographic trends, land use strategies, and processes of environmental adaptation. Within Africa, archaeological Big Data can contribute to the study of the spread of agriculture, domesticated species, and specific artefacts and technologies, as well as their ecological impacts. Although reviews addressing these topics are available for different parts of the continent, existing mid-late Holocene archaeology datasets have yet to be compiled into a central, open-access, standardized informatic-oriented dataset. Here we present *Wanyika*, a dataset of scientific dates from archaeological sites in eastern Africa spanning almost 7 millennia, from ~5000 BCE to 1800 CE. This dataset compiles published scientific dates and associated botanical, faunal, iron, and ceramic finds from sites in Kenya, Tanzania, the Comoros Islands, and Madagascar. The records also include data for megafauna extinctions in Madagascar. We describe the spatiotemporal coverage of the dataset, how the data were collected, the structure of the dataset, and the applied quality control measures.

## Introduction

Despite its importance in major models of human demographic and linguistic expansion, agricultural spread, and Indian Ocean trade, the Holocene archaeological record of eastern Africa remains largely unsynthesized and a source of significant debate^1–8^. Effectively addressing major archaeological questions, such as whether late Holocene changes in the archaeological record of eastern Africa reflect climate-driven migrations of different human populations, and applying archaeological information to contemporary socio-environmental challenges, especially using new archaeoinformatics approaches, requires the compilation of existing datasets on past human-environment interactions^2,9–13^. As it stands, much of the relevant archaeological information on eastern Africa’s past is dispersed across publications of varying accessibility, precluding or inhibiting the kinds of analyses that are currently being applied in other regions, and that can shed light on key research questions and debates^9,14–23^.

**Fig. 1:**
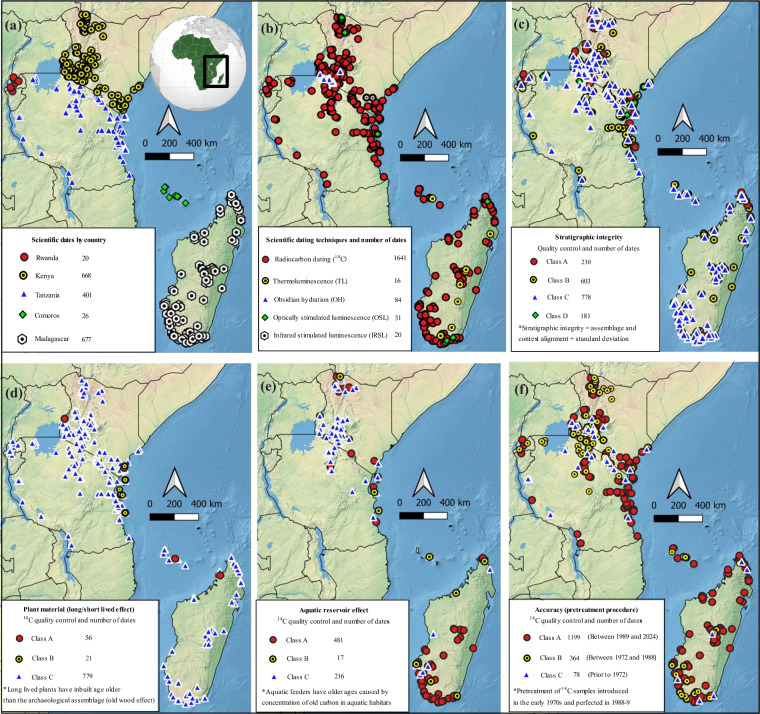
Maps of eastern Africa highlighting the disparities in spatial distribution of scientific dates by (**a**) country and (**b**) scientific dating technique (^14^C, OSL and IRSL, TL, and OH); and distribution of dates based on the four quality control measures: (**c**) stratigraphic integrity and reliability, (**d**) ^14^C dates from plant material, (**e**) ^14^C dates from faunal material, and (**f**) ^14^C dates accuracy in pre-treatment procedure. Note: Class A dates are the most reliable. We used QGIS version 3.28.9^545^, Natural Earth^546^, CSV data files^547^, Adobe Photoshop^548^, Inkscape app^549^, to generate Fig. 1 maps.

Since the early 20^th^ century, there has been significant growth in available archaeological data for the mid-to-late Holocene in eastern Africa^24–30^. These data are the outcome of the application of a variety of approaches, including excavation and survey, as well as archaeobotanical, zooarchaeological, geoarchaeological, isotopic, palaeoproteomic, coring, and remote sensing methods^30–42^. However, available records have yet to be compiled into a standardized dataset format. Here, we present *Wanyika*^43^, a dataset of scientific dates and associated archaeological records from mid-late Holocene sites covering four countries (plus a selection of sites in Rwanda) in eastern Africa (Fig. 1). The dataset focuses on these four countries as they possess some of the best documented archaeological records in eastern Africa for this time period, in particular as a result of the application of radiocarbon dating. *Wanyika*^43^ is an informatics-oriented dataset that draws together data spanning almost seven millennia, from 5000 BCE to 1800 CE. The Bantu term ‘*Wanyika*’ translates as “people of the wilderness” and is used to refer to all inland ethnic groups of eastern Africa, as well as those that migrated to the littoral islands and Madagascar^44–46^. The associated archaeological records include spatiotemporal data pertaining to botanical, faunal, iron, and ceramic finds from published archaeological sites, in addition to several unpublished sites, across key regions of mainland and island eastern Africa. We have included iron and ceramic finds because they are closely—although not exclusively—associated with the spread of food production in eastern Africa^3,5,27,47–65^. Ceramic finds are vital because the ceramic styles of hunter-gatherers, pastoralists, and farmers are different^1,5,47,51,52,58,60,62,64,66–79^. Records for megafaunal persistence and coexistence with humans in Madagascar are also included^30,80–108^. Rather than a comprehensive overview, the Wanyika dataset is a preliminary work that serves as a foundation for future research.

### Archaeology in eastern Africa

Eastern Africa’s past is marked by significant transformations in economic organization, food production, trade, social connections, and climatic conditions^5,6,39,40,109–115^. Pastoral communities entered eastern Africa from the north by 4,000 BCE, but pastoralism spread gradually and heterogeneously across the region over the next few thousand years^116–118^. The origins of crop farming are less clear^119^. While scholars argue that farming may have spread into eastern Africa by 500 BCE, the first evidence for domesticated crops does not appear until the period 300 BCE - 250 CE^27,72^. Early farmers and herders also relied on hunting and gathering, as well as on trade with Indigenous forager populations^5,6,10,25,53,70,120–123^. Eventually, forms of food production involving diverse indigenous crops and exogenous livestock coalesced in eastern Africa before spreading to other parts of the continent^26,53,60,124–128^. There remain major debates as to the role that the migration of different cultural groups^3,30,31,36,63,114,122,129–133^ and major changes in climatic and environmental conditions^134–137^ played in these processes. During the 1^st^ and 2^nd^ millennia CE, eastern Africa became part of expanding proto-global exchange networks across the Indian Ocean, with crops, animals, spices, material culture and ideas arriving from Southwest Asia, South Asia and Southeast Asia^1,26,55,124,138–143^.

Major research gaps and unresolved issues persist in eastern African archaeology. For example, archaeological research is unevenly distributed across the region^60,128^. Extensive areas of eastern Africa have seen minimal research, while researchers have only selectively applied archaeological science methods. This means that large swathes of interior eastern Africa lack archaeobotanical evidence for the origin and dispersal of crops, while coastal and island zones have only recently and patchily been investigated^5,6,28,64,119,144–148^. Furthermore, a significant percentage of excavated sites lack scientific dates^1,26,30,57,125,147–171^. Almost half of the faunal remains recovered from eastern African archaeological sites have not been identified to the species level^70,72,73,172–178^, and the potential of residue analysis and micro-CT scanning in ceramic studies, and of digital archaeology more generally, have yet to be fully explored^33,41,175,179–183^. Meanwhile, debate continues regarding the involvement of pastoralists in the dispersal of sorghum and finger millet to the interior of eastern Africa^5,25,59,111,114,119,131,184^. Contention also surrounds the spread of livestock, early farming, iron production, and pottery manufacture, particularly to the coast and islands^1,53,119,171,185–194^, and the role of human colonisation and activity in megafaunal extinctions in Madagascar^30,83,85,195^. In part, this debate stems from unresolved regional chronologies that would otherwise aid understanding of the origin and dispersal of food production in eastern Africa and the spread of iron-working and ceramic traditions, warranting the revisiting and reanalysis of existing datasets^30,82,186,187,196^.

These deficiencies mean that many research questions in eastern African archaeology cannot at present be adequately addressed. These include questions surrounding the role of diverse populations in the different stages of eastern Africa’s deep-time history, as well as the spread of diverse languages, species, and technologies. Improved datasets are also required to understand the impact of changing land use on local and regional environments and the role of anthropogenic activities in shaping biodiversity^30,82,83,87^. Disentangling the complex fisher-forager-pastoralist-farmer interactions in various geographical settings also requires improved data^62–64,68,74,153,187,194,197–201^. Here, in order to begin to more systematically address these critical lacunae at the regional scale, we compile the first archaeological dataset of absolute dates along, with associated botanical and faunal remains, and iron and ceramic artefacts. By highlighting existing data gaps, and paving the way for assembling large, regional-scale datasets, we aim to facilitate initiatives aiming to address major research questions in eastern Africa, and to apply past data to current and future challenges.

## Methods

### Sampling strategy

The *Wanyika*^43^ dataset covers sites located in Kenya, Tanzania, Comoros, and Madagascar that date to the period between c. 5,000 BCE and 1,800 CE (Fig. 1). The dataset addresses all scientifically dated sites in these countries, providing details of available dates, as well as information about associated crop, faunal, iron, and ceramic finds. In addition, selected Rwandan sites with early evidence for domesticated crops and iron artefacts are included in light of their importance to the study of farming dispersals in eastern Africa^27,47,72,119,184^.

The four countries currently covered by the database, which represent an initial sample of the 18 countries that define eastern Africa^202,203^, were selected because of their significant contributions to understanding past human-environment interactions in this broader region. Compared to the northern and southern regions of eastern Africa, the countries of the coast (Kenya and Tanzania) and islands (Comoros and Madagascar) have well-documented Holocene archaeological records and are at the heart of key archaeological debates surrounding early farming dispersals and biological exchange mediated by long-distance mobility and cross-cultural interaction^3,5,8,16,30,32,53,55,70,72–74,82,124,131,132,153,195,198–201,204–214^. Over the last 25 years, the application of archaeological science approaches in these countries has resulted in the recovery and identification of an increased number of archaeobotanical and zooarchaeological finds to genus or species level, and has also produced significant numbers of chronometric dates and material culture remains^7,30,31,33,34,36–38,42,53,55,62,63,67,68,70–72,74,80–83,85,87,91,93,96,98,103,117,124,130,144,152,172,174,178,185–187,190–193,195,200,209,215–272^.

Rwandan sites have been included in the dataset because they provide some of the earliest documented evidence of domesticated crops in eastern Africa, and also lie within the hypothesized dispersal route of farmers into the interior of eastern Africa^16,47,127,207,273–279^. Evidence for the dispersal of crops across interior eastern Africa is limited; apart from eight Rwandan sites, there are only two sites with archaeobotanical records (Kakapel and Deloraine in Kenya) in the interior^72,119,184^. These Rwandan sites have produced archaeobotanical remains associated with Urewe ceramics and iron, which are critical to the interpretation of archaeological assemblages in the interior of eastern Africa^27,47^. The Rwandan region is also hypothesized to have served as a gateway and dispersal point for farming communities migrating into the interior of eastern Africa through either the Mount Elgon or Lake Tanganyika region.

Whilst the dataset does not represent the entirety of eastern Africa, we present Wanyika as a foundational repository that provides a systematic framework for future expansion of eastern African archaeological datasets. As a cautionary note to users of the dataset, we have included a limited set of sites (only sites with scientific dates), which biases estimates about the effect sizes in the existing eastern African archaeological data. The selection of sites with scientific dates might lead to misleading inference at a broad spatiotemporal scale about the question of unravelling the complex interactions of farmers, pastoralists, and foragers in different geographical settings in eastern Africa.

### Demarcation of country regions and vegetation cover

To facilitate the exploration of geographical patterns in the data (e.g., Table 3), Kenya, Tanzania and Madagascar were sub-divided into smaller sub-regions (‘Country Regions’), e.g., southwestern, northwestern (see Table 1). These divisions are widely used in the archaeological literature^1,58,59,61,66,94,112,116,118,153,155,159,187,190,199,204,209,239,280–319^, but have not been formally constrained before using geographical coordinates. The boundaries used in this study were defined as follows. Mainland Kenya is divided into four broadly equal-sized regions demarcated by latitude 0.5° and longitude 37.7°, with the hinterland, coast and islands demarcated as the fifth region. Likewise, mainland Tanzania is divided into four regions using latitude −6° and longitude 35°, with the hinterland, coast and islands demarcated as the fifth region. Madagascar is also divided into four almost equal-sized regions using latitude -19° and longitude 47°. The predominant vegetation cover for each site and region have also been included^13,320–324^. These are divided into six categories, including forest/wood/grassland mosaic, montane forest, coastal forest mosaic, dry coastal wooded grassland, dry northern wooded grassland, and dry southern wooded grassland.

**Table 1:** Demarcation of regions by country boundaries and site bibliographic reference citations, predominant vegetation cover, and approximate boundaries by geographical coordinates of a delimiting rectangle.

Regions and site bibliographic reference citations	Predominant vegetation cover	Latitude	Longitude
Rwanda (No regions)^27,47–50,273,278,356–358^	Forest/wood/grassland mosaic	−1.1°, −1.1° −2.8°, −2.6°	29.2°, 30.7° 28.8°, 30.9°
Comoros (No regions)^53,55,60,264,359–365^	Coastal forest mosaic	−11.3°, −12.8° −11.9°, −13.2°	43.2°, 45.4° 43.0°, 45.3°
Northeastern Kenya^176,288,366,367^	Dry northern wooded grassland	5.4°, 4.1° 0.5°, 0.5°	37.7°, 41.9° 37.7°, 41.1°
Northwestern Kenya^31,61,66,67,72,75,116–118,175,177,204,215,220–229,232,233,283,291–293,312,337,368–400^	Dry northern wooded grassland Forest/wood/grassland mosaic	5.7°, 5.4° 0.5, 0.5°	33.4°, 37.7° 33.8°, 37.7°
Southeastern Kenya^144,172,401^	Dry northern wooded grassland	0.5°, 0.5° −3.2°, −5.0°	37.7°, 41.1° 37.7°, 39.4°
Southwestern Kenya^5,24,28,29,31,36,37,42,57–59,61–65,68,70,73–75,77,78,121,123,130,132,156,159,174,177,184,197,204,205,212,217,234,235,247,280,309,311,343,371,391,402–450^	Dry northern wooded grassland Montane forest Forest/wood/grassland mosaic	0.5°, 0.5° −1.1°, −3.2°	33.8°, 37.7° 33.8°, 37.7°
Hinterland, Coastal and Island Kenya^1,8,26,34,55,56,128,154,172,188,189,216,218,251,294,297,310,451–463^	Coastal forest mosaic	−1.4°, −2.0° −4.7°, −5.0°	41.4°, 41.8° 39.1°, 39.6°
Northeastern Tanzania^36,38,70,71,79,128,145,147,148,155,200,204,230,239,240,245,252–254,256–262,287,300,317,430,464–477^	Forest/wood/grassland mosaic Dry northern wooded grassland	−1.8°, −4.7° −6°, −5.8°	35.0°, 39.1° 35.0°, 38.4°
Northwestern Tanzania^59,60,146,204,205,410,470,478–484^	Forest/wood/grassland mosaic Dry northern wooded grassland	−0.8°, −0.9° −5.6°, −6.0°	29.5°, 34.1° 29.4°, 35.0°
Southeastern Tanzania^300,305,307,485,486^	Dry southern wooded grassland	−6.0°, −5.7° −11.6°, −10.9°	35.0°, 38.3° 35.0°, 39.8°
Southwestern Tanzania^157,314–316,319,487,488^	Dry southern wooded grassland	−5.8°, −6° −8.2°, −11.6°	29.6°, 35.0° 30.7°, 35.0°
Hinterland, Coastal and Island Tanzania^1,37,53–55,124,125,142,152–155,185–189,199,209,217,218,236–238,241,243,244,246–248,250,263,295,296,298,300–308,318,455,485,489–514^	Coastal forest mosaic	−4.9°, −4.9° −10.9°, −10.9°	38.8°, 40.1° 39.8°, 41.1°
Northeastern Madagascar^55,80,82,87,90,94,104,106,129,190–193,270,290,313,374,515–523^	Coastal forest mosaic	−11.8°, −12.8° −19.0°, −19.0°	47.0°, 51.9° 47.0°, 49.1°
Northwestern Madagascar^80,82,87,97,100,267,269,313,524,525^	Dry coastal wooded grassland	−15.3°, −12.0° −19.0°, −19.0°	44.7°, 47.0° 43.7°, 47.0°
Southeastern Madagascar^82,87,94,97,522,526^	Coastal forest mosaic	−19.0°, −19.0° −25.2°, −25.6°	47.0°, 49.2° 47.0°, 47.2°
Southwestern Madagascar^30,80–89,91–94,96–105,107,195,265,266,268,271,272,371,517,527–543^	Dry coastal wooded grassland	−19.0°, −19.0° −24.7°, −25.2°	43.7°, 47.0° 43.2°, 47.0°

### Data collection and deposition

**Fig. 2:**
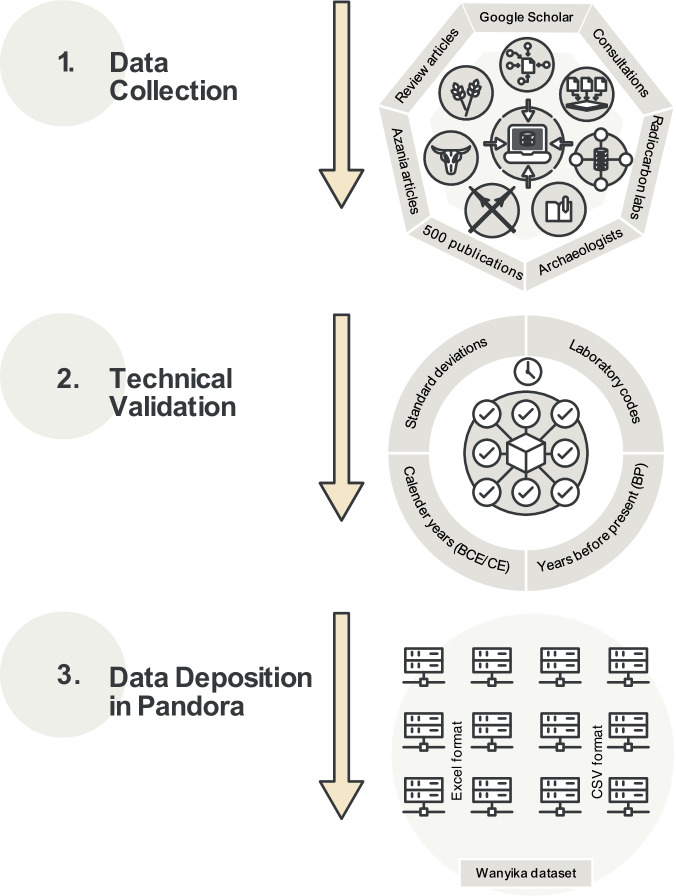
Flow chart summarizing the construction of the Wanyika dataset^43^.

Data collection followed the workflow summarized in Fig. 2. The authors drew upon more than 500 scientific publications as data sources based on citations in major review articles and other seminal works on the study regions^1,5,25,30,53,55,60,82,83,87,111,124,128,131,138^. The scientific search engine Google Scholar was employed to locate further articles using a combination of keywords such as specific country/region names, “archaeology”, “scientific dates”, “archaeobotany”, “zooarchaeology”, “iron”, and “ceramics”. The authors also screened all available volumes of *Azania: Archaeological Research in Africa* (1967 to 2023) in order to collect further scientific dates and information about associated archaeological finds. For just over half of the published dates, the associated assemblage evidence, i.e. archaeobotanical, zooarchaeological and artefactual evidence, was obtained from separately published specialist articles. Where it was necessary to clarify data issues and locate missing publication data, the original publication authors or expert archaeologists working in eastern Africa were consulted. Radiocarbon dating laboratories were also contacted to provide missing details on published dates and dated material. Based on the references given in review articles and other seminal publications, and on discussions with researchers familiar with the study region, we estimate that at least 90% of the published scientific dates and associated archaeological records from the sampled eastern Africa countries are captured in the *Wanyika* dataset^43^.

Upon completion, the *Wanyika* dataset was deposited in the Pandora data platform. Pandora is a multi-language, web-based, data management platform, where data communities self-manage membership and self-curate data in various formats^202^.

### Scientific dates and calibration

We employ the term “scientific date” to refer to a date that is determined using scientific dating methods to establish the age of an artefact, feature, and/or site. These methods provide a quantifiable measure of time with an associated margin of error. Five types of scientific dating techniques were reported in the publications that were consulted to compile the *Wanyika* dataset^94,144,280,325–328^: radiocarbon dating (^14^C), optically stimulated luminescence (OSL), infrared stimulated luminescence (IRSL), thermoluminescence (TL), and obsidian hydration (OH) (Fig. 1(b)). Radiocarbon dates are calculated based on the abundance of the ^14^C isotope in samples (e.g., archaeobotanical remains or collagen extracted from zooarchaeological remains)^325,329^. Luminescence dating methods such as OSL, IRSL, and TL determine the last time mineral grains were exposed to sunlight or sufficiently high temperatures^144,325,328^. Finally, OH dating measures obsidian water absorption to determine the age of an object. Although OH dating can be used to determine absolute or relative dates, only absolute dates (which can be summarized in intervals of calendar years, as opposed to just older or younger than^280,325^) are recorded in the *Wanyika* dataset. Relative dates based on material culture typologies such as ceramics and beads are also not included in our dataset^1,127,277^.

The scientific date of each sample appears in three formats: one showing uncalibrated years before present (BP) in two fields expressing the date BP and standard deviation (SD), another showing calibrated calendar years (BCE and CE) in three fields expressing a 95% probability range plus a mean, and finally one showing calibrated years BP also in three fields expressing a 95% probability range plus a mean. The reference year for uncalibrated ^14^C and OH dates is 1950. For ^14^C, the uncalibrated or conventional dates are reported so that their calibration can be revised once calibration curves are updated.

In the case of OSL, IRLS, and TL dates, BP is presented here relative to publication year rather than the conventional BP (Before Present, i.e., 1950). We note that this may differ from sample recovery or sample analysis date, which may represent the actual reference year. However, this choice is seldom reported and we do not anticipate a significant offset between the actual reference year and publication date. Luminescence dating techniques were first introduced in the 1960s, setting a highly conservative upper bound for a potential offset between publication year and the unknown reference year of c. 50 years^144,325,328,330–333^.

The ^14^C dates were calibrated using the most recent calibration curves (IntCal20 and SHCal20) and the Bayesian chronological software OxCal v.4.4^334–336^. For samples located to the south of latitude -17° (the southernmost limit of the Intertropical Convergence Zone; ITCZ), only SHCal20 was used in the calibration process. For samples to the north of this latitude, it was necessary to account for the mixing of northern and southern curves along the ITCZ^337,338^. In these cases, we calibrated radiocarbon measurements using a mixed IntCal20 and SHCal20 curve with an unknown level of mixing (flat prior between 0 and 100 in OxCal).

Radiocarbon calibrations for aquatic samples followed a protocol similar to that described in Goldstein *et al*.^12^. Briefly, for marine shells the marine ΔR radiocarbon offset was calculated around each burial location (radius of 100 km) for a ΔR smoothed surface generated using data from the Marine Reservoir Correction dataset^339^ and the Bayesian model AverageR^340,341^. Radiocarbon calibration into calendar dates was done using the Bayesian chronological software OxCal v4.4^334^. To calibrate a single inland shell, found in the vicinity of the Turkana salt lake, an inland ΔR mean value of 250 ^14^C yrs relative the IntCal20 calibration curve^335^ was used following Beck *et al*.^342^ with an assigned uncertainty of ± 100 ^14^C yrs.

As stated previously, for OSL, IRLS, TL, dates, the calibration process consisted of subtracting the reported BP date from the publication year while for OH the reported BP date was subtracted from 1950^64,117,144,185,270,278,343^. The dates were converted into calendar years and reported as a 95% probability calendar range (BCE/CE).

For all methods, and when available, the dataset also includes the description of the dated sample type and the taxonomic identification. Questionable measurements are flagged under a notes field in the dataset records (e.g., when there were discrepancies among different measurements/dating methods or dates did not agree with known chronological boundaries for a cultural layer). Problematic dates often originated from mixed/unclear contexts^72,186,187,196^.

### Quality control

We developed four chronometric quality control criteria for the scientific dates and used them to grade dates in the dataset into classes. The first criterion is based on a combination of stratigraphic integrity and reliability and is applied to all dates in the dataset to grade them into four classes (Class A–D, with A being the most secure and reliable, and D being the least secure and reliable) (Fig. 1 and Tables 2, 3). The other three criteria were only applied to ^14^C dates and are based on (i) whether the date was obtained on short or long lived plant material (i.e., the potential presence of an old wood effect)^337^, (ii) the possible presence of an aquatic radiocarbon reservoir effect^344^, and (iii) the accuracy of the chronometric determination (Fig. 1 and Table 4). In each of these cases, dates were assigned into three classes (Class A–C), again with A being the most reliable and C being the least reliable. A description of how the quality control criteria were applied is provided below.

**Table 2:** Date qualities defining classes in the stratigraphic integrity and reliability quality control measure.

Class	Qualities
Class A	Dates have a secure stratigraphic association and an SD of 25 or less.
Class B	Dates either: (1) have a secure stratigraphic association and an SD between 26–100; or (2) have a questionable stratigraphic association and an SD of 25 or less.
Class C	Dates either: (1) have a secure stratigraphic association and an SD of 101 or above; (2) have a questionable stratigraphic association and an SD between 26–100; or (3) have unreported context details and an SD of 50 or less.
Class D	Dates either: (1) have a questionable stratigraphic association and an SD of 101 or above; or (2) have unreported context details and an SD of 51 or above.

**Table 3:** Country and regional summaries of dating methods and quality control (chronometric hygiene based on stratigraphic integrity and reliability of the dates), highlighting the scarcity of Class A dates in the Wanyika database.

	Total Number of dates	Dating Method					Quality Control (Chronometric Hygiene)			
		Number of dates					Class A-D			
		Radiocarbon	OSL	OH	TL	IRSL	A	B	C	D
**COUNTRY TOTALS**										
Rwanda	20	20	0	0	0	0	**3**	14	2	1
Comoros	26	25	0	0	1	0	**7**	10	8	1
Kenya	668	556	8	84	0	20	**99**	161	279	129
Tanzania	401	397	4	0	0	0	**61**	224	94	22
Madagascar	677	643	19	0	15	0	**74**	190	387	26
**GRAND TOTAL**	**1792**	**1641**	**31**	**84**	**16**	**20**	**244**	**599**	**770**	**179**
**REGIONAL TOTALS**										
Northeastern Kenya	5	5	0	0	0	0	**0**	3	2	0
Northwestern Kenya	135	127	8	0	0	0	**55**	29	44	7
Southeastern Kenya	59	39	0	0	0	20	**0**	9	15	35
Southwestern Kenya	394	310	0	84	0	0	**39**	89	211	55
Hinterland, Coastal and Island Kenya	75	75	0	0	0	0	**5**	31	7	32
Northeastern Tanzania	140	140	0	0	0	0	**12**	82	39	7
Northwestern Tanzania	51	51	0	0	0	0	**0**	16	24	11
Southeastern Tanzania	6	6	0	0	0	0	**0**	6	0	0
Southwestern Tanzania	9	9	0	0	0	0	**0**	4	5	0
Hinterland, Coastal and Island Tanzania	195	191	4	0	0	0	**49**	116	26	4
Comoros	26	25	0	0	1	0	**7**	10	8	1
Northeastern Madagascar	142	127	6	0	9	0	**46**	46	45	5
Northwestern Madagascar	28	28	0	0	0	0	**0**	11	14	3
Southeastern Madagascar	26	24	0	0	2	0	**0**	20	4	2
Southwestern Madagascar	481	464	13	0	4	0	**28**	113	324	16
**GRAND TOTAL**	**1772**	**1621**	**31**	**84**	**16**	**20**	**241**	**585**	**768**	**178**

See Table 1 for information on demarcation of country regions.

**Table 4:** Grading scheme for ^14^C dates for plant and aquatic materials, and accuracy of chronological determination.

Short- vs long-lived plant material	Grade
Sample from taxonomically identified short lived plant parts: Annual seeds, leaves and twigs	**A**
Sample from taxonomically identified long lived plant parts: Perennial seeds, leaves and twigs	**B**
Unknown/unreported: Taxonomically unidentified plant parts: Charcoal	**C**
**Aquatic radiocarbon reservoir effects**	
Fully terrestrial sample	**A**
Aquatic sample	**B**
Unknown/humans or other animals that may have consumed aquatic foods	**C**
**Accuracy of chronological determinations (Pre-treatment protocol)**	
Published after 1988. The ultrafiltration method and AMS were introduced around 1988.	**A**
Published between 1972 and 1988. The Longin method was introduced in 1971.	**B**
Samples published prior to 1972	**C**

### Stratigraphic integrity and reliability grading

The stratigraphic integrity for each date was evaluated by assigning points to dates based on the following system (Column BI). Six points were given to a date if the authors of the original publication indicated that it was reasonably well associated with its archaeological context (i.e. the stratigraphic integrity was not questioned). Three points were given to a date that was questioned by either the original authors or in a subsequent publication, for example because the date is more recent than that of the overlying context, or older than that of the underlying context, possibly due to bioturbation. Finally, zero points were awarded to a date that had no contextual information, which is recorded as ‘Unreported’ in Column J. Radiocarbon dates on ratite eggshell and dates obtained using obsidian hydration (OH), which have been shown to be unreliable dating methods^30,64,280,337,343^, were treated as questioned dates and awarded three points, unless they had no context details in which case they were awarded zero points. Notes on the stratigraphic integrity of a date are found in Column BH of the dataset. We did not undertake any evaluation of the stratigraphic integrity of individual dates other that which has been reported in the published literature.

The reliability of a scientific date was evaluated on the basis of its standard deviation (Column N), with six, four, two and zero points assigned to dates with SDs between 0–25, 26–50, 51–100 and 101–650, respectively.

Subsequently, the points assigned to each date for their stratigraphic integrity and reliability were added and divided by two to give a mean score, which was used to generate Class A-D dates with mean scores of 6, 4–5.5, 2–3.5 and 0–1.5, respectively. The application of the grading system produced comparable class qualities (Table 2). Table 3 summarizes the number of dates based on country and region, dating method, and the stratigraphic integrity and reliability quality control grades.

The dataset^43^ records from Kakapel (Entry IDs 217 to 244), Gogo Falls (Entry IDs 203 to 216) provide good examples of the grading system. Kakapel has 28 dates, 23 of which are Class A and five of which are Class B. The Class B dates are different because they have SDs between 25–50. Gogo Falls has 14 dates, of which none are Class A because all of the dates have SDs above 60. It has eight Class B dates, four Class C dates and two Class D dates. Gogo Falls also provides examples of bioturbation and OH dating challenges (e.g., Entry ID 204, which is an OH date with an SD of 115 and therefore classified as Class D). The process by which ratite eggshell dates were classified are illustrated by examples from Andakatomena and Tony-Velondriake in Madagascar. A radiocarbon date from Andakatomena on *Aepyornis* eggshell (Entry ID 1124) was considered questionable owing to the use of an unreliable dating method and had an SD of 25 or less, so was classified as Class B. A similar date from Tony-Velondriake, also on *Aepyornis* eggshell (Entry ID 1120) and with an SD of 25 or less, was classified as Class C owing to its lack of contextual information.

### Radiocarbon (^14^C) date grading

Radiocarbon dates underwent additional chronometric hygiene using three different criteria (Table 4). The first two criteria consider the in-built age of the sample at death, which varies with the type of material selected for dating. In the case of plant materials, age offsets may be present due to the selection of long-lived wood materials, often referred to as ‘the old wood effect’^337,345^. Dates obtained on taxonomically identified short-lived plant parts (e.g., annual seeds, leaves, twigs) were graded as Class A, dates obtained on taxonomically identified long-lived plant parts were graded as Class B, and dates where the taxonomic identification of plant parts was not reported were graded as Class C (see Column BJ in the dataset).

The second criterion considered the potential for an age offset owing to an aquatic radiocarbon reservoir effect^344^, caused by the uptake of ^14^C-depleted carbon by a marine or freshwater organism or the inclusion of such organisms into human or animal diets^344,346–348^ (Column BK). The degree to which aquatic radiocarbon reservoirs affect radiocarbon dates varies depending on local environmental conditions (which can also change over time), as well as variations between species owing to their habitat and dietary preferences^349^. We took a coarse approach to this problem, assigning samples with a fully terrestrial diet to Class A, aquatic samples to Class B, and samples that have an unreported origin or derive from humans or animals that may have consumed aquatic foods to Class C. A more detailed analysis of the original publications to assess, for example, whether local radiocarbon reservoir offsets were calculated for dates or the likelihood of aquatic diets using stable isotope data was not undertaken.

The third criterion considers the accuracy of a chronological determination based on the pre-treatment method used and its effectiveness at removing inorganic and organic contaminants, which may skew an age determination. Pre-treatment methods were not recorded in the dataset, and indeed, have rarely been reported alongside radiocarbon determinations in the study region. We employed a coarse filter based on the publication date of the assay (Column BL), working with the knowledge that the accuracy of the ^14^C dating technique for bone organics was refined in 1971, when the Longin collagen extraction method was introduced^350^, and in 1988, when ultrafiltration was introduced^351^. It was also around this time that AMS become an increasingly routine technique in radiocarbon dating of bone and charcoal samples^329^. Dates reported after 1988, as well as those subjected to chromatographic methods (e.g. XAD, amino acid isolation using HPLC), were assigned to Class A, those reported between 1972 and 1988 were assigned to Class B, and those published prior to 1972 were assigned to Class C.

## Data Records

The *Wanyika* dataset^43^ has 75 fields organized within eight major categories (Table 5). We provide definitions of these 75 column fields below. *Wanyika*^43^ is a spatiotemporal, flat-file dataset in which each row of the dataset represents a single scientific date associated with archaeological records. The total number of dataset records is 1792, each associated with one of 422 sites. The presence of domesticated crop, faunal, iron, and/or ceramic finds is marked by “Yes” in the specific cell, while absence is represented by a blank cell.

**Table 5:** Data categories and field details. See the section below for definitions of column fields.

Column categories	Data entry details
Site details & metadata	Each row is identified by a unique entry ID, site ID, name, country, region, vegetation cover, latitude and longitude coordinates, trench/site notes and context details
Date	Date type, laboratory code number, mean BP date and standard deviation (uncalibrated in the case of radiocarbon measurements), minimum and maximum ranges and mean BCE/CE calendar dates, dated material, dated taxon, ceramic and regional cultural phase
Archaeobotanical	Presence of major African and exotic crops
Zooarchaeological	Presence of wild tetrapods, avian, aquatic, and indeterminate fauna, domesticates, human remains, introduced fauna, and extinct megafauna in Madagascar
Artefacts	Presence of iron artefacts and ceramics
Notes	Important qualitative site data
Quality control measures (Chronometric hygiene)	Applied to all dates: Based on stratigraphic integrity and reliability Applied to ^14^C dates: Based on dated material and refined dating technique
Reference identification	Source references given in the *American Journal of Biological Anthropology* citation format and their Digital Object Identifier (DOI). Five fields are used to cite multiple references.

The dataset is made available in both Excel and CSV formats via the repository *Wanyika* (https://pandoradata.earth/dataset/wanyika) within the AfriArch data community on the Pandora data platform https://pandoradata.earth/organization/afriarch^202^. The upload is the peer-reviewed version of the dataset and will remain static.

### Definition of column fields

The column fields are identified by alphabet letters, followed by column title and definition of what the column represents.

A - Entry ID: This number represents a row containing the details of a particular date entry.

B - Site ID: This is a database number assigned to a site. Each site has a unique number. All entries from the same site have the same number.

C - Site Name: Refers to the name of the archaeological site.

D - Country: Name of the country where a site is located (Rwanda, Kenya, Tanzania, Comoros, and Madagascar).

E - Country Region: Name of the region where a site is located within a country, defined by administrative boundaries and latitude/longitude. Information on demarcation of country regions is provided in Table 1.

F – Vegetation cover: Describes the dominant vegetation of the site^13,320,322,323,352^. Includes six categories: Forest/wood/grassland mosaic, montane forest, coastal forest mosaic, dry coastal wooded grassland, dry northern wooded grassland and dry southern wooded grassland.

G - Latitude: Provides the site’s GIS coordinates for the latitude expressed in decimal degrees relative to WGS84^353^.

H - Longitude: Provides the site’s GIS coordinates for the longitude expressed in decimal degrees relative to WGS84^353^.

I - Trench/Site Notes: Provides the excavators’ code number details for the excavation trench, unit, square, quadrant, test pit, feature (e.g., burial, furnace, habitation), and any other unique attributes that define a site (e.g., single component or stratified site short/long term occupation).

J - Context: This includes the context number and/or measurement in cm, layer, level, and stratum. Provides details that define time and space, matrix and provenience.

K - Date Type: Indicates the type of scientific dating method used: ^14^C, OSL, IRSL, TL or OH.

L - Labcode: This is a code number assigned by the host dating laboratory to the sample used to produce a scientific date. Mostly represented by letters and numbers, where letters are the lab designation and the number the unique sample number, for example OXA-14500.

M - Date BP: Uncalibrated date Before Present.

N - Date BP SD: Standard deviation for the uncalibrated date Before Present.

O - Min Chronology (Calibrated BCE/CE): Minimum calibrated date in calendar years (Before Common Era and Common Era).

P - Max Chronology (Calibrated BCE/CE): Maximum calibrated date in calendar years (Before Common Era and Common Era).

Q - Mean Chronology (Calibrated BCE/CE): Mean calibrated date in calendar years (Before Common Era and Common Era), Minimum + Maximum / by 2 (calibrated date in calendar years).

R - Min Chronology (Calibrated BP - 1950): Minimum calibrated date Before Present (1950 minus Min Chronology calibrated BCE/CE).

S - Max Chronology (Calibrated BP - 1950): Maximum calibrated date Before Present (1950 minus Max Chronology calibrated BCE/CE).

T - Mean Chronology (Calibrated BP - 1950): Mean calibrated date Before Present (Minimum + Maximum / by 2 (Calibrated date Before Present).

U - Dated Material: Type of material/sample that was used to produce the date e.g., charcoal, ceramics, bone collagen, etc.

V - Dated Taxon: Provides the lowest order taxonomic group (species, genus, family, order or kingdom) for plant and animal remains (seeds, charred wood, bone/tooth collagen and apatite) used in dating.

W to AP - Archaeobotanical Information: Columns provide archaeobotanical information. A “Yes or blank cell” indicates the presence or absence of a specific crop. Represented African crops include finger millet (*Eleusine coracana*), pearl millet (*Pennisetum glaucum*), sorghum (*Sorghum bicolor*), cowpea (*Vigna unguiculata*), hyacinth bean (*Lablab purpureus*), Fig (*Ficus sp*.) and baobab (*Adansonia digitata*); exotic crops include rice (*Oryza sativa*), wheat (*Triticum aestivum*), coconut (*Cocos nucifera*), mung bean (*Vigna radiata*), pea (*Pisum*), red date (*Ziziphus jujuba*), and cotton (*Gossypium sp*.).

AQ to BB - Zooarchaeological Information: Columns provide zooarchaeological information for wild and pastoral species (but excluding introduced commensals like cat, dog, black rat). A “Yes or blank cell” indicates the presence or absence of a specific faunal taxon. Faunal taxa include wild tetrapods, avian, aquatic, and indeterminate fauna, as well as pastoral animals like bovids, cattle (*Bos taurus/indicus*), sheep (*Ovis aries*) and goat (*Capra hircus*), indeterminate Ovis/Capra, camel (*Camelus dromedary*), donkey (*Equus asinus*) and chicken (*Gallus gallus*). Directly dated remains of extinct megafauna in Madagascar (ratites, lemuroids, reptiles and non-primate mammals) and other translocated fauna (commensals cat, dog, black rat) are only recorded in the “Dated Taxon” column (column U).

BC to BD - Iron Smelting and Use: “Yes or blank cell” represents the presence or absence of evidence for iron smelting and use.

BE - Ceramics: “Yes or blank cell” represents the presence or absence of evidence for ceramics.

BF - Ceramic Phase (Pottery Ware): Lists the name/s of ceramic style/s associated with the row data. These include 25 major ceramic/pottery tradition/wares in eastern Africa listed in Table 6.

**Table 6:** Period and ceramic tradition associated with regional cultural phases.

Regional Phase	Estimated period	Ceramic tradition
Prehistoric (Madagascar)	Pre-600 CE^30,87^	—
Late Stone Age (LSA)	6000 BCE – 500 CE^74,438,544^	Kansyore, Eburran, Lowasera
Savanna Pastoral Neolithic (SPN)	5000 BCE – 750 CE^6,74,122^	Nderit, Ileret, Maringishu, Narosura, Akira
Elmenteitan Pastoral Neolithic (EPN)	1400 BCE – 750 CE^6,122^	Elmenteitan
Pastoral Iron Age (PIA)	600 CE – 1800 CE^122^	Turkwell, Lanet, Kisima, and Oldorotua
Early Iron Age (EIA)	500 BCE – 700 CE^53^	Urewe, Lelesu, Kwale, Tana, Maore, Kilimanjaro, Swahili, Asian, Rezoky, Beropitika, Andaro, and Tranovato
Middle Iron Age (EIA)	701 CE – 1000 CE^53^	
Later Iron Age (LIA)	1001CE – 1800 CE^53^	

BG - Regional Cultural Phase (Eastern Africa): Lists the regional cultural phase represented by the recorded cultural assemblage. These includes, (a) Prehistoric (applicable for Madagascar), LSA (Late Stone Age), PN (Pastoral Neolithic), the latter divided into SPN (Savanna Pastoral Neolithic) and EPN (Elmenteitan Neolithic), PIA (Pastoral Iron Age); and (b) EIA (Early Iron Age), MIA (Middle Iron Age), LIA (Late Iron Age). See Table 6 for the time period and ceramic tradition associated with each regional cultural phase.

BH - General Notes: Provides important notes about the site/date. These include notes that were used for quality control assessment, for example notes on questionable dates and anomalies as a result of bioturbation.

BI - Mean Grade Chrono Hygiene 1 and 2: Refers to stratigraphic integrity grades tabulated from stratigraphic integrity plus standard deviation (SD) mean scores to produce Class A-D dates. See more details in the section on quality control.

BJ - Chrono Hygiene 3: Grade based on the possibility that the ^14^C date is affected by long life span plant material (long/short). See Table 4

BK - Chrono Hygiene 4: Grade based on the possibility of the dated material being affected by aquatic ^14^C reservoir. See Table 4

BL - Chrono Hygiene 5: Grade based on the accuracy of chronological determinations (pre-treatment protocol) for ^14^C dates. See Table 4

BM - Date of Publication: refers to the earliest year in which the date was published. This year is used to calculate the grades for “Chrono Hygiene 5: Accuracy (pre-treatment) C_14_ dates” and the BP dates for OSL, IRSL, and TL (the primary publications used the year of excavation as ‘the present’, we use the publication year).

BN to BW - References and DOI 1-5: Provides the bibliographic references and Digital Object Identifier (DOI) or the Uniform Resource Locator (URL) for the sources of data.

## Technical Validation

The authors retrieved the data recorded in this dataset primarily from peer-reviewed scientific journals, books, dissertations, monographs, and site reports written by established researchers working in eastern Africa. In several cases, data are also included from ongoing projects awaiting publication. Only dates reported in uncalibrated years before present, having standard deviations and laboratory codes, are included in the dataset. We carried out frequent checks throughout our database construction process and a final comprehensive check was completed towards the end of the process to remove duplicate records and other errors. Approximately 20 dates were excluded for lack of uncalibrated dates (reported in BCE/CE only), SD or laboratory code. For example, two dates from Engaruka and Kuumbi Cave (Tanzania) were excluded for lack of standard deviations^185,187,258^. A unique case that involved four dates from the Serengeti (Tanzania) being published as Takwa dates (Kenya) was noted^59,204,354^. These dates were also excluded from the dataset. The author of the Takwa report (page 4, first sentence) acknowledges the error^354^. Personal correspondence with Geochron Laboratories also confirmed that these dates belong to the Serengeti.

## Usage Notes

The FAIR data principles promote good data management practices by encouraging scientists to make their datasets ‘findable’, ‘accessible’, ‘interoperable’, and ‘reusable’^355^. The *Wanyika* dataset^43^, as a member of the Afriarch community hosted on the Pandora platform, adopts these principles. The public availability afforded by Pandora’s web presence makes the data findable and enables accessibility. *Wanyika*^43^ promotes data reuse by explicating the results of the quality control analysis and by being open to collaborations that can update the dataset as new information becomes available. Ongoing development of the Pandora platform includes Natural Language Model SQL (Standard Query Language) programming that grants interoperability to datasets such as *Wanyika*^43^, which furthermore has the potential for integration into semantically interoperable dataset systems such as MAEASaM (Mapping Africa’s Endangered Archaeological Sites and Monuments).

## Data Availability

We have used four codes to calibrate the ^14^C dates in OxCal for (1) terrestrial samples between -18° and 6° latitude, (2) terrestrial samples below -18° latitude, (3) marine samples, (4) freshwater samples (only applicable to a sample above 0.5° latitude). These codes are provided below. Terrestrial samples located between -18° and 6° latitude Plot(‘terrestrial samples’) { Curve(‘IntCal20,‘IntCal20.14c’); Curve(‘SHCal’,‘SHCal20.14c’); Mix_Curves(‘Mixed_terrestrial_curves’,‘IntCal20’, ‘SHCal20’,U(0,100)); R_Date(‘LAB_CODE’,14C_MES,14C_UNC); } Terrestrial samples below -18° latitude Plot(‘terrestrial samples’) { Curve(‘SHCal’,‘SHCal20.14c’); R_Date(‘LAB_CODE’,14C_MES,14C_UNC); } Marine samples Plot(marine samples’) { Curve(‘Marine20’, ‘Marine20.14c’); Delta_R(‘LocalMarine’, MARINE_DR, MARINE_DR_UNC);} R_Date(‘LAB_CODE’,14C_MES,14C_UNC); } Freshwater samples (only applicable to a sample above 0.5° latitude Plot(‘freshwater samples’) { Curve(‘IntCal20,‘IntCal20.14c’); Delta_R(‘LocalFreshwater’, FRESHWATER_DR, FRESHWATER_DR_UNC);} R_Date(‘LAB_CODE’,14C_MES,14C_UNC); } Definition of terms for OxCal code for calibration of radiocarbon measurements LAB_CODE stands for radiocarbon lab code 14C_MES stands for the uncalibrated radiocarbon measurement in years BP 14C_UNC stands for the 1-sigma error associated to 14C_MES MARINE_DR stands for local marine ΔR value MARINE_DR_UNC stands for the 1-sigma error associated to MARINE_DR FRESHWATER_DR stands for local freshwater ΔR value FRESHWATER_DR_UNC stands for the 1-sigma error associated to FRESHWATER_DR
